# Dynamic Alternative Polyadenylation during *Litopenaeus Vannamei* Metamorphosis Development

**DOI:** 10.3390/genes15070837

**Published:** 2024-06-26

**Authors:** Xueqin Yang, Xiuli Chen, Chengzhang Liu, Zezhong Wang, Wei Lei, Qiangyong Li, Yongzhen Zhao, Xia Wang

**Affiliations:** 1 China (Guangxi)-ASEAN Key Laboratory of Comprehensive Exploitation and Utilization of Aquatic Germplasm Resources, Ministry of Agriculture and Rural Affairs, Guangxi Academy of Fishery Sciences, Nanning 530021, China; yangxueqin1109@163.com (X.Y.); chenxiuli2001@163.com (X.C.); 2 College of Animal Science and Technology, Northwest A&F University, Yangling 712100, China; wangzz2024@126.com; 3 Key Laboratory of Aquaculture Genetic and Breeding and Healthy Aquaculture of Guangxi, Guangxi Academy of Fishery Sciences, Nanning 530021, China; lqy_cz@163.com; 4 CAS Key Laboratory of Experimental Marine Biology, Institute of Oceanology, Chinese Academy of Sciences, Qingdao 266071, China; liu@qdio.ac.cn; 5 Department of Pharmaceutical and Graduate Life Sciences, College of Pharmacy, Natural & Health Sciences, Manchester University, Fort Wayne, IN 46845, USA; wlei@manchester.edu

**Keywords:** alternative polyadenylation, dynamic regulation, *Litopenaeus vannamei*, metamorphosis, miRNA

## Abstract

As an important mechanism in the post-transcriptional regulation of eukaryotic gene expression, alternative polyadenylation (APA) plays a key role in biological processes such as cell proliferation and differentiation. However, the role and dynamic pattern of APA during *Litopenaeus vannamei* metamorphosis are poorly understood. Here, RNA-seq data covering from the embryo to the maturation (16 time points) of *L. vannamei* were utilized. We identified 247 differentially expressed APA events between early and adult stages, and through fuzzy mean clustering analysis, we discovered five dynamic APA patterns. Among them, the gradual elongation of the 3′UTR is the major APA pattern that changes over time, and its genes are enriched in the pathways of protein and energy metabolism. Finally, we constructed mRNA-miRNA and PPI networks and detected several central miRNAs that may regulate *L. vannamei* development. Our results revealed the complex APA mechanisms in *L. vannamei* metamorphosis, shedding new light on post-transcriptional regulation of crustacean metamorphosis.

## 1. Introduction

Metamorphosis is a process by which immature individuals undergo dramatic anatomical and physiological changes to develop into adults. [1]. Metamorphosis is a critical evolutionary and developmental transition that is often accompanied by behavioral, feeding, ecological, and physiological changes. [2]. In most marine invertebrates, metamorphosis transforms a developing larva into the adult benthic stage, triggered by external environmental cues. Apparently, this metamorphosis, which allows larvae to recognize and respond to signals from different benthic substrates, ensures that they can settle in habitats suitable for their growth and survival [3,4].

In *L. vannamei*, a member of the Crustacea subphylum, metamorphosis encompasses the development from zygote to adult, passing through distinct stages of embryo, nauplius, zoea, mysis, post-larvae, and adult [5]. *L. vannamei* metamorphosis is influenced by a variety of factors, including temperature, food, and salinity. This fundamental shift necessitates extensive physiological changes in the organism, driven by a cascade of intricate signaling pathways that precisely control the expression of specific genes [6,7].

In multicellular eukaryotes, alternative polyadenylation (APA) is an important post-transcriptional regulatory mechanism, playing a crucial role in development and tissue-specific functions [8,9]. It is a process of genes leveraging different cleavage/polyadenylation (C/P) sites (polyA sites) to generate different transcripts’ isoforms with varying 3′ untranslated region (3′UTR) lengths [10,11]. Alternative 3′UTR isoforms interact with different microRNAs (miRNAs) and RNA-binding proteins [9] to regulate gene expression at the post-transcriptional level [12]. These include changes in mRNA localization, degradation rates, and translation efficiency [13]. It has been reported that in different tissues or developmental stages of humans and mice, genes exhibit transcripts with varying lengths of 3′UTRs [14,15,16]. For example, 3′UTRs undergo extensive changes during mammalian embryonic development, with 3′UTRs shortening in proliferating cell types such as blood [17] and lengthening in neuronal cells [18,19]. APA patterns vary considerably at different developmental stages [20,21], as shown by recent studies. For example, in mouse embryonic development, the switching between proximal and distal isoforms is temporally specific, indicating the dynamic role of APA during development [22]. However, the mechanism of APA during the metamorphosis of *L. vannamei* remains elusive.

To this end, we collected public RNA sequencing (RNA-Seq) data of 16 different time points during dynamic developmental stages of *L. vannamei* [5,23]. We systematically analyzed their APA landscape and observed that metabolism-related genes underwent the progressive lengthening of 3′UTRs during development. By further investigation of APA regulatory factors and their impact on miRNA regulation, several central miRNAs are identified as potentially regulating *L. vannamei* development. In conclusion, our systematic analysis of APA pattern variations during metamorphosis suggests potential developmental mechanisms governing APA usage in *L. vannamei*. Our results shed new light on APA regulation and the metamorphic development of crustaceans.

## 2. Materials and Methods

### 2.1. Data Collection

We retrieved publicly available RNA-Seq data for 16 dynamic developmental stages of *L. vannamei* (zygote to adult) from NCBI under accession numbers SRP094135 [23] and SRP061180 [5]. This dataset encompasses nine sequential early developmental stages (zygote, blastula, gastrula, limb bud embryo, larva in membrane, nauplius, zoea, mysis, and post-larvae) and seven adult stages (pre-molt D0, D1, D2, D3, and D4, and post-molt P1 and P2).

### 2.2. APA Analysis

After the removal of low-quality reads and adapters, all reads were aligned against ASM378908v1 using HISAT2 (version 2.2.1). Subsequently, the resultant BAM files served as input for DaPars2 (Masamha et al., 2014; Xia et al., 2014b) to de novel predict proximal polyA sites and quantify the APA using the percentage of distal polyA site usage index (PDUI). For a given transcript, the lower the PDUI value was, the more proximal polyA site was utilized, and vice versa. Meanwhile, if the PDUI value was missed in more than 5 time points, its APA was discarded. Then, we identified differences in the 3′UTR between the two stages using paired t-tests. Differential APA events were defined as having an adjusted *p*-value (Benjamini–Hochberg) below 0.05 and an absolute change in |ΔPDUI| ≥ 0.1 between the two stages. Next, we clustered the median-centered PDUI of differential APA events using a fuzzy c-means approach [24].

### 2.3. Motif Feature Analysis

Sequences within 50 nt upstream and downstream of proximal and distal polyA sites were separately extracted for each APA event using BEDTools (version 2.31.1) [25]. Subsequently, sequence features for the proximal and distal sites were predicted respectively using DREME (version 5.5.5) [26].

### 2.4. Principal Component Analysis

To identify distinct stages during *L. vannamei* development, we performed PCA analysis on the median-centered PDUI values of the top 500 APA events ranked by standard deviation using the R function ‘prcomp’ in the R package stats.

### 2.5. Differential Gene Expression Analysis

We used salmon (version 1.10.1) [27] to estimate transcript levels and generate a transcript per million (TPM) matrix, retaining genes expressed in at least 8 samples with TPM ≥ 0.1. We then performed Wilcoxon signed-rank tests on the TPM values of early developmental stage and adult stage samples, identifying differentially expressed genes (DEGs) (FDR < 0.05). Afterward, DEGs and the genes of differential APA events were intersected, and a Venn diagram was drawn.

### 2.6. Function Enrichment Analysis

To characterize the potentially different mechanisms of APA lengthening and APA shortening before and after *L. vannamei* metamorphosis, we used the differential APA genes screened previously to perform the GO function enrichment analysis using the web of g:Profiler (https://biit.cs.ut.ee/gprofiler/gost, accessed on 14 November 2023). GO terms which satisfied FDR < 0.05 were defined as significantly enriched terms in differentially expressed genes. Meanwhile, KEGG pathway analysis was performed using KAAS (https://www.genome.jp/tools/kaas/, accessed on 20 November 2023) to identify significantly enriched metabolic pathways [28]. Enrichment analysis and visualization were performed using the R package clusterProfiler [29]. The significance of KEGG analysis was set to a Bonferroni-corrected *p*-value < 0.05.

### 2.7. mRNA-miRNA and PPI Network Construction

We leveraged Rnahybrid and miRanda to predict miRNA binding sites within alternative 3′UTR regions of differentially expressed APA events. Only the common binding sites predicted by the above two algorithms were selected. Then, the degree of the mRNA-miRNA targeting relationship was calculated using the R package. The top 25 genes were selected to build an mRNA-miRNA regulatory network, and Cytoscape was used to visualize it. Subsequently, OrthoDB (https://www.orthodb.org/, accessed on 5 December 2023) was used to identify homologous genes for differentially APA events, and a total of 166 homologous genes were found and we constructed miRNA-APA networks by performing protein–protein interaction (PPI) analyses on genes within each APA event identified by Metascape (https://metascape.org/gp/index.html#/main/step1, accessed on 10 December 2023) [30], and visualized using Cytoscape (version 3.10.1).

## 3. Results

### 3.1. Global Patterns of APA Events during L. vannamei Development

**Figure 1:**
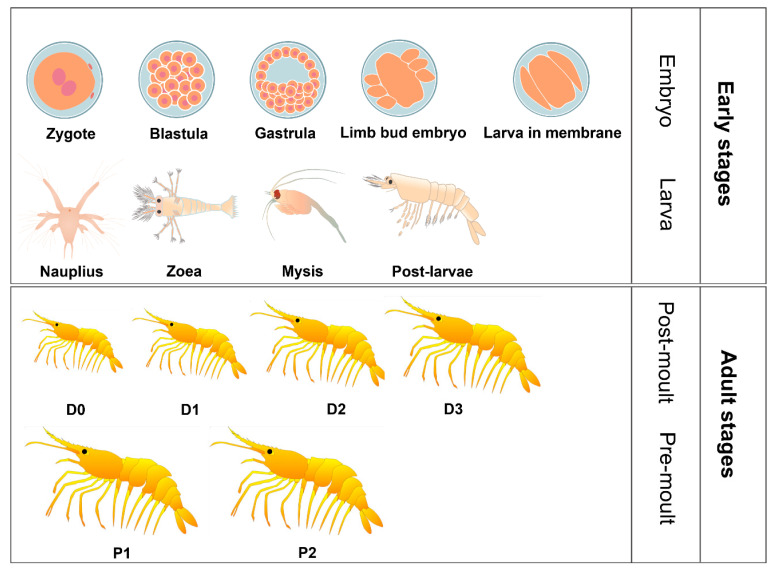
Early and adult stages of *L. vannamei* development.

To investigate dynamic APA profiles during *L. vannamei* development, we analyzed publicly available RNA-Seq data. Previous research divided the *L. vannamei* growth cycle into 16 characteristic stages based on morphological and physiological features and sequenced RNA from each stage [23] (Figure 1). To generate a comprehensive APA profile, we employed the DaPars2 [11] algorithm, a widely used tool for APA analysis, to predict the percentage of PDUI for each transcript. This analysis yielded 2957 APA events across 2822 known genes.

**Figure 2:**
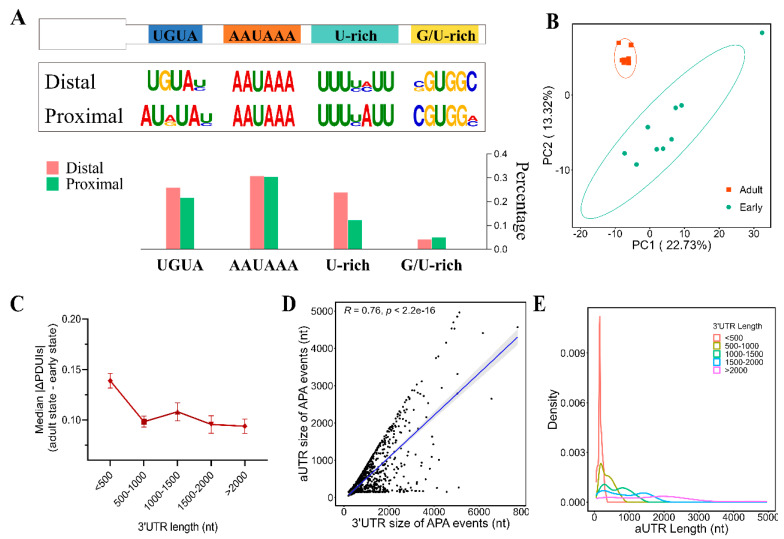
Global landscape of alternative polyadenylation (APA) during *L. vannamei* development. (**A**) Enriched motifs around proximal and distal polyA sites of APA events. The barplot shows the percentage of each motif feature. (**B**) PCA analysis result. (**C**) 3′UTR size (*x*-axis) and aUTR size (*y*-axis) of each APA event. The correlation coefficient (r) and *p*-value were calculated by Pearson analysis. (**D**) Median-centered |ΔPDUI| values between early and adult stages for *L. vannamei* to represent global APA variation. |ΔPDUI| values were classified into different bins of 3′UTR sizes. (**E**) Density plot of aUTR sizes classified by different bins of 3′UTR sizes.

Building upon previous research that has illuminated a diverse array of regulatory cis-elements responsible for polyA site recognition, we sought to further characterize these elements within our specific dataset [31]. To this end, we extracted sequences extending ± 50 nucleotides from both proximal and distal polyA sites within identified APA events and subjected them to motif enrichment analysis using DREME [26]. This analysis revealed the presence of well-established cleavage and polyadenylation cis-elements, including AAUAAA, UGUA, G/U-rich, and U-rich sequences, flanking both distal and proximal polyA sites (Figure 2A).

Across time points, average PDUI values of APA events hovered around 0.5, indicating that about half of the APA events utilized distal polyA sites (Table S1). Notably, a number of APA events exhibited dynamic changes during *L. vannamei* development, including a pronounced surge after the post-larval stage. PCA applied to PDUI values effectively classified samples into two distinct groups: early stage and adult stage (Figure 2B).

We further investigated the relationship between APA changes and 3′UTR length. Analyzing median-centered |ΔPDUI| for each APA event, we assessed global APA shifts during development from early stages (zygote, blastula, gastrula, limb bud embryo, larva in membrane, nauplius, zoea, mysis, and post-larvae) to adult stages (pre-molt D0, D1, D2, D3, and D4, and post-molt P1 and P2). Dividing 3′UTR lengths into intervals, we observed that genes with lengths of 500 nt or less displayed the most pronounced |ΔPDUI| changes during development (Figure 2C). It is noteworthy that the 3′UTR length, defined as the distance between distal and proximal polyA sites, is primarily 300 nt in APA events. Furthermore, we observed a strong positive correlation between 3′UTR and aUTR lengths (R^2^ = 0.76) (Figure 2D,E). These findings suggest that genes with shorter 3′UTRs are more likely to be subjected to developmental regulation via APA.

### 3.2. Characterization of APA Events during L. vannamei Development

**Figure 3:**
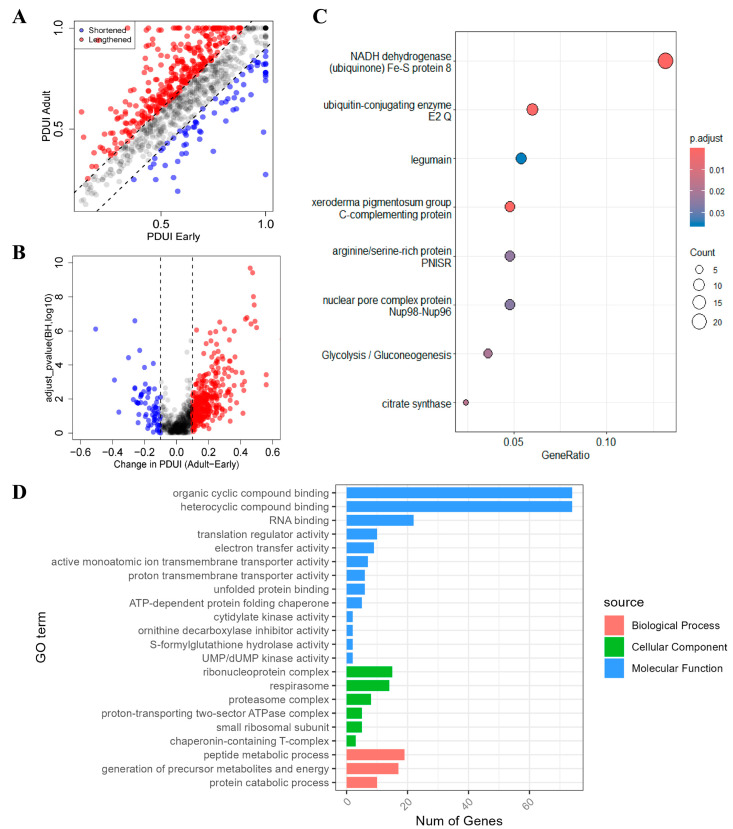
Characterization of APA events during *L. vannamei* development. (**A**) Scatterplot of PDUIs in early (*x*-axis) and adult (*y*-axis) samples from the *L. vannamei* cohort. Significantly (adjusted *p*-value < 0.05 and |ΔPDUI| > 0.1) shortened and lengthened transcripts are indicated in red and blue, respectively, whereas those below the threshold are gray. (**B**) Volcano plot showing the significantly altered APA events in the *L. vannamei* cohort. (**C**) Function annotation of differential APA events. (**D**) Differential APA events of KEGG enrichment analysis.

**Figure 4:**
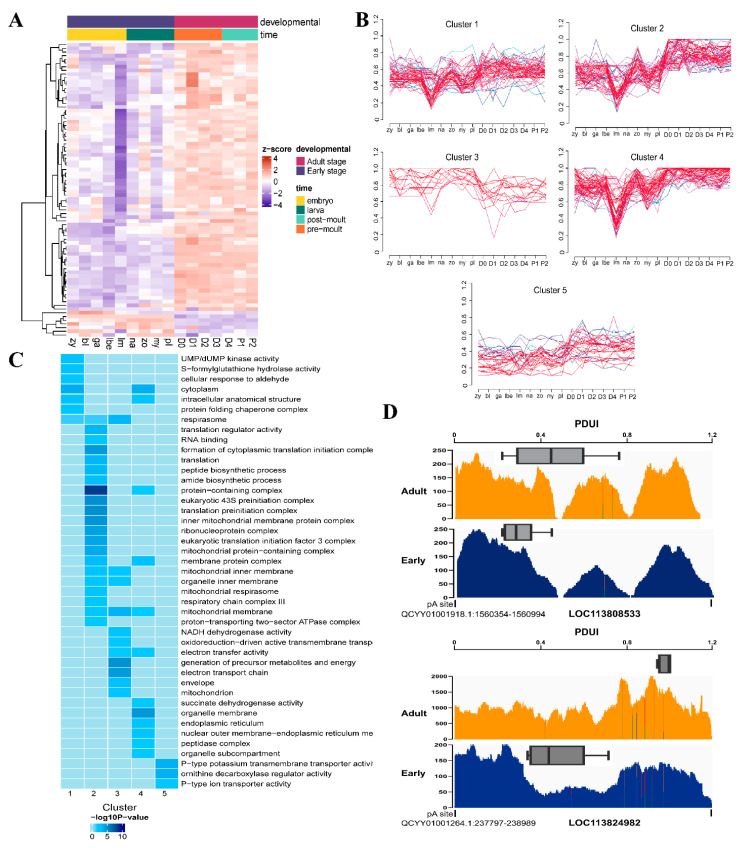
Dynamics of APA changes during *L. vannamei* development. (**A**) Heatmap of differential APA events. Each APA event was normalized by Z-score. (**B**) Tracks of different APA patterns during L. vannamei development. (**C**) Heatmap of gene enrichment results of APA patterns. (**D**) Read coverage of *LOC113808533* and *LOC113824982* visualized by IGV. The *y*-axis represents time points during cardiomyocyte differentiation. The proximal polyA site was obtained from DaPars results.

To comprehensively delineate alterations in APA events between early and adult developmental stages, we meticulously analyzed differences in PDUI values between the two groups. Employing stringent thresholds of |PDUIadult − PDUIearly| > 0.1 and an adjusted *p*-value below 0.05, we successfully identified a total of 247 differential APA events. Among these, 212 exhibited 3′UTR lengthening, while 35 displayed 3′UTR shortening (Figure 3A,B and Figure 4A).

Given the prevalence of APA changes in our analysis, we next sought to gain deeper insights into the potential functions of these differentially regulated transcripts. Through GO and KEGG enrichment analyses, we identified significant overrepresentation of pathways associated with protein structure and function, signaling, and respiratory chain energy metabolism, such as organic cyclic compound binding, electron transfer activity, chaperonin-containing T-complex, and NADH dehydrogenase (ubiquinone) Fe-S protein 8 (Figure 3C,D). Detailed information on the enrichment analysis is provided in Table S2.

### 3.3. Dynamics of APA Events during L. vannamei Development

Next, we examined the changes in APA during *L. vannamei* development. To meticulously delineate the dynamic patterns of APA events during *L. vannamei* development, we harnessed the power of fuzzy c-means cluster analysis using Mfuzz [24] to scrutinize the median PDUI values of differential APA events (Figure 4B). This comprehensive analysis unveiled five distinct APA patterns, some of which defied categorization into simple monotonic trends. The key characteristics of these patterns are as follows: (1) Cluster 1: the PDUI trend remains relatively flat throughout development, with a notable decrease at the larva in membrane stage. (2) Cluster 2: the PDUI trend exhibits an overall increase during development, punctuated by rapid declines at the larva in membrane and mysis stages, followed by subsequent increases at the zoea and D0 stages. (3) Cluster 3: PDUI decline is exclusively observed at the D0 stage. (4) Cluster 4: development marked by rapid PDUI drops at the larva in membrane and mysis stages. (5) Cluster 5: the PDUI trend remains stable throughout development. Among these patterns, Cluster 5 harbored the largest number of APA events (*n* = 59).

Functional enrichment analysis revealed pathway associations with distinct APA patterns (Figure 4C). GO terms related to protein synthesis, such as translation regulator activity, the translation preinitiation complex, and the protein-containing complex, and GO terms related to the mitochondrial respiratory chain, such as NADH dehydrogenase activity, the electron transport chain, and the generation of precursor metabolites and energy, were significantly enriched in Clusters 2 and 3. Additionally, terms associated with intracellular energy anabolism, respirasome, and the mitochondrial membrane were present in multiple clusters.

### 3.4. Differential Expression and Alternative Polyadenylation of Cleavage and Polyadenylation Factors during L. vannamei Development

**Figure 5:**
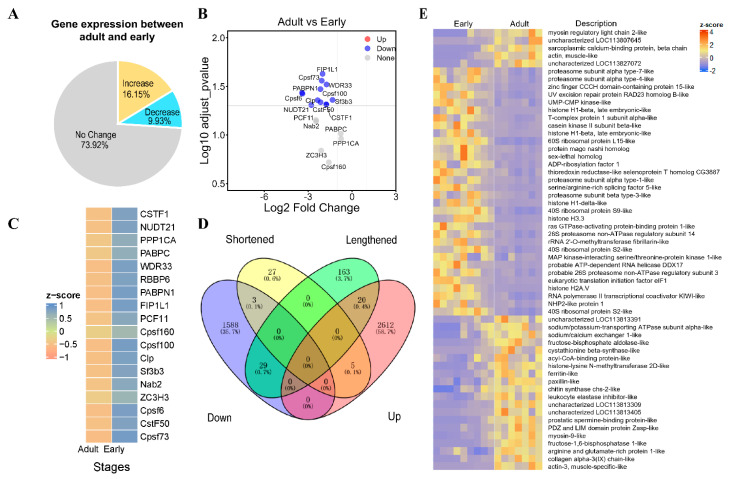
Differential expression and cleavage and polyadenylation factors during *L. vannamei* development. (**A**) Pie chart displaying expression changes of APA genes in *L. vannamei* development. (**B**) Differential expression volcano plots of key transcription factors such as CPSF, CstF, CFI, and CFII at early developmental and adult stages. (**C**) Heatmap displaying expression changes of core polyA regulators. (**D**) APA differential events and overlapping genes in DEGs. Overlapping genes shared by more than one are represented in the area of intersection between 2 circles. (**E**) The expression of overlapping genes between DEGs and APA differential events.

To further investigate the post-transcriptional regulation of APA, we analyzed the differentially expressed genes between adult and early stages, identifying a total of 4259 differentially expressed mRNAs, with 2638 upregulated and 1621 downregulated (Figure 5A). In addition, recent studies have shown that changes in the polyadenylation mechanism can directly affect its own regulatory components, leading to shortening or elongation of the 3′UTR [32]. This mechanism involves key transcription factors such as *CPSF*, *CstF*, *CFI*, and *CFII* [33]. We found that the expression of most core polyA regulatory factors was significantly reduced in the adult stage (Figure 5B,C). We hypothesized that the expression patterns of these transcription factors are linked to the regulation of gene expression before and after *L. vannamei* metamorphosis. Subsequently, 57 genes were found to overlap between DEGs and differentially APA events (Figure 5D,E). Among others, LOC113815123 (actin-3, muscle-specific-like), LOC113815118 (actin, muscle-like), LOC113823552 (myosin regulatory light chain 2-like), LOC113810364 (sarcoplasmic calcium-binding protein, β chain), and LOC113829812 (myosin-9-like), which are annotated in the reference genome as being related to muscle growth and development, have significantly higher expression in the adult stage than in the early developmental stage, and their 3′UTR length is regulated by APA.

### 3.5. mRNA-miRNA and PPI Network Construction Network during L. vannamei Development

**Figure 6:**
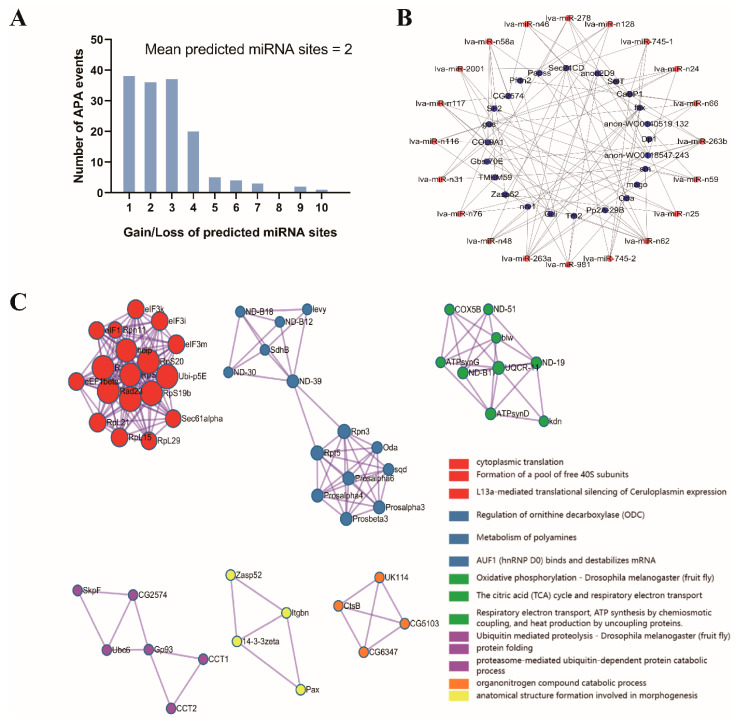
Statistics of miRNAs in aUTRs and network. (**A**) Distribution of miRNA binding sites in aUTRs of differential APA events. (**B**) Differential APA event mRNA-miRNA interaction network analysis. (**C**) The PPI network constructed from the homologous genes of genes with differential APA events. The lines represent the APA gene–gene with STRING PPI > 0.7.

Given that 3′UTR change can disrupt functional elements like miRNA binding sites [34], we investigated potential changes in miRNA binding sites associated with APA events during development. We predicted *L. vannamei* miRNA binding sites within aUTRs, revealing 454 binding sites could be affected (gained or lost) by the 39 APA events (Figure 6A). Based on prediction, lva-miR-n62, lva-miR-263b, and lva-miR-263a emerged as the top three hub regulatory miRNAs within the mRNA-miRNA(aUTR) network (Figure 6B). Furthermore, the differential APA genes were mapped to the protein–protein interactions (PPI) from the STRING database (Figure 6C). A total of 56 nodes and 207 edges were involved in the PPI network and were enriched and analyzed according to GO pathways and processes, with cytoplasmic translation scoring the highest *p*-value.

## 4. Discussion

*L. vannamei*, the world’s most commonly cultured shrimp, has attracted significant investment in genetic and genomic resources to drive healthy farming practices [35,36]. Like other metamorphosing animals, *L. vannamei* undergoes a meticulously orchestrated and intricate metamorphosis, requiring precise co-regulation at multiple transcriptional, post-transcriptional, and epigenetic levels. To dissect the potential contribution of APA to this crucial developmental phase, we employed RNA-seq data across distinct developmental stages, spanning embryonic to post-mature individuals, to comprehensively map APA events in developing shrimp. During *L. vannamei* development, we identified distinct APA events exhibiting stage-specific regulation, suggesting their potential involvement in modulating gene expression patterns. As *L. vannamei* develop, the prevalence of distal PAS usage in the 3′UTR leads to an overall increase in RNA length. The extended regions of the 3′UTR may harbor some miRNA/RNA-binding protein sites, which may be involved in the regulation of target gene expression. Therefore, cleavage and polyadenylation events are dynamically regulated throughout *L. vannamei* development, highlighting their significance as a key regulatory mechanism coordinating this intricate metamorphic process.

Many marine invertebrates undergo a rapid transition from plankton to benthos [37]. During this metamorphosis, intercellular signaling pathways relay specific external cues (e.g., temperature, light) which lead to modulated gene expression profiles [6]. Furthermore, transcripts involved in key physiological processes like energy acquisition, stress adaptation, immune defense, and cell death signaling have been linked to metamorphic transitions in various studies [38,39]. Our analysis revealed enrichment of differential APA events in pathways is associated with protein and energy metabolism, prominently within protein catabolic processes, precursor metabolite and energy generation, and translation regulator activity (Figure 3C,D). This may be related to the increased need for biosynthesis and metabolite interconversion during metamorphosis [40,41]. Low expression of genes associated with energy production across both embryonic and nauplius stages is consistent with the limited internal nutrient stores of the animals at this developmental phase [37]. *L. vannamei* primarily feed on other zooplankton during early developmental stages, transitioning to a diet of both zooplankton and phytoplankton as adults [42]. To adapt to shifting dietary quality and anisotropic organ growth (e.g., liver/muscle), they utilize diverse metabolic pathways for efficient energy production and metabolite interconversion throughout their development [43]. Furthermore, adapting to low salinity and osmolality environments necessitates increased energy expenditure [44]. This demand can be met through diverse metabolic pathways, including mitochondrial and sugar metabolism, fatty acid metabolism, and amino acid metabolism. Notably in the present study, GO was enriched to the pathway associated with proton transmembrane transporter activity, which involves changes in the 3′UTR of many mitochondria-related genes (Table S2), such as cytochrome c oxidase subunit 6A (*COX6A1*). Previous studies have shown that the expression of many mitochondrial genes is altered in adult Chinese sturgeons exposed to salinity changes [45]. Cytochromes b, c, and P450 are all components of the electron transport chain in the inner mitochondrial membrane, where oxidative phosphorylation and the generation of an electrochemical proton gradient take place, with the latter serving as the driving force for ATP synthesis [46]. In addition, KEGG was significantly enriched in the biological processes associated with respiratory chain electron transport (Figure 3D). Among them, NADH dehydrogenase (ubiquinone) Fe-S protein 8 is a protein located in the mitochondrial inner membrane and is part of the NADH dehydrogenase complex I [47]. NADH dehydrogenase complex I is one of the most important electron transport chain complexes in cells, responsible for transferring electrons from NADH to coenzyme Q [48]. These results demonstrate that APA plays a critical role in the response to a variety of pathways and signals. However, further experimental validation is needed to confirm the rapid response of APA to small molecules.

The selection of APA sites involves core cleavage and polyadenylation factors binding to specific pre-mRNA sequences, influenced by various regulatory factors like transcriptional activity, pre-mRNA cis-regulatory elements, and protein binding [49]. The CPSF complex interacts with PAPOLA, CPSF1 (FIP1L1/FIP1), and WDR33 [50,51]. Mechanistically, CPSF-73 interacts with PAPOLA to mediate the cleavage of pre-miRNA and the addition of a poly(A) tail [52]. CPSF-160 functions as a platform to recruit WDR33 to the appropriate conformation for PAS binding [53]. The cleavage stimulation factor (CstF) complex comprises the subunits CstF50 (CSTF1), CstF64 (CSTF2), and CstF77 (CSTF3) [54], and primarily recognizes U/G-rich DSEs through CstF64 [55]. The assembly of these complexes leads to their binding to pre-mRNA. Subsequently, poly(A) polymerase (PAP) is recruited to the cleavage site and adds a poly(A) tail. [52]. In this study, we observed an overall lengthening of 3′UTRs in the adult stage (Figure 3A,B), and the expression of these core factors was significantly lower compared to the early developmental stage (Figure 5B,C). Unsurprisingly, even minute changes in the concentration of any of these protein factors can impact 3′ end processing and modulate the relative abundance of alternative 3′UTR isoforms by regulating APA. For example, knockdown of PCF11, which previously promoted proximal PAS usage, resulted in the upregulation of distal PAS usage in C2C12 myoblast mouse cells [56]. Additionally, it downregulates the expression of short genes, particularly those harboring weaker PASs [57]. While core polyA regulators are known to govern APA, the precise molecular mechanisms and environmental cues by which they control 3′ end cleavage site selection to drive distinct mRNA isoforms during normal differentiation and development remain elusive.

To further investigate the regulatory mechanisms of APA in the development of *L. vannamei* metamorphosis, we focused on the function of overlapping genes in the Venn diagram (Figure 5D). We found that the extension or shortening of 3′UTRs of muscle-related genes during shrimp metamorphosis may be regulated by APA, thereby affecting gene expression. During arthropod metamorphosis, skeletal muscles are remodeled, with larval muscles atrophying and adult muscles growing and regenerating. Gene expression profiles change during this process [58]. Previous studies have shown that during the remodeling of larval to adult dorsal muscles in *Xenopus laevis* metamorphosis, myosin heavy chain and myosin light chain isoforms undergo transformation [59], and significant changes in the levels of 12 muscle proteins have been identified [60]. Therefore, the dynamic regulation of APA may have synchronized the differential expression of muscle remodeling-related genes during shrimp metamorphosis.

In addition, progressive lengthening of the 3′UTR is the primary mode of APA during the entire developmental process of shrimp. This global extension of the 3′UTR, regulated by APA, has been observed during the embryonic development of mice and zebrafish [61,62], suggesting that it might play a universal role in various aspects of animal growth and development, including morphogenesis, differentiation, and proliferation. Furthermore, 3′UTRs lengthen and increase the use of intron polyA sites, leading to the acquisition of multiple miRNA target sites during development [34]. Longer 3′UTRs may contain more binding sites, while shorter 3′UTRs are subject to more stringent regulation. Our constructed mRNA-miRNA and PPI network revealed a series of miRNAs regulating *L. vannamei* growth and development, among which the miR-263a/b appears to have multiple functions. For example, in molting animals, miR-263b negatively regulates the expression of *LanA*, a key component of the brain barrier, and is involved in the regulatory mechanisms of tissue remodeling and growth during the transition from larva to pupa [63]. Other miRNAs such as lva-miR-n62, lva-miR-n66, and lva-miR-n48 can be used as novel regulators of *L. vannamei* metamorphosis. To sum up, our findings demonstrate that widespread APA events rewire miRNA-targeting landscapes. These targeting alterations provide novel insights into the functional implications of miRNAs during the metamorphic development of *L. vannamei*.

## 5. Conclusions

In this work, we mapped the dynamic APA landscape during *L. vannamei* development, revealing unique APA patterns associated with specific developmental periods, including the lengthening of 3′UTRs throughout development. On the other hand, we found that differentially APA genes were mainly concentrated in protein metabolism and energy metabolism pathways. Furthermore, we found that APA post-transcriptionally regulates the extension or shortening of 3′UTRs of muscle growth and development-related genes, affecting the expression of genes, and is involved in muscle remodeling during *L. vannamei* metamorphosis. Finally, we constructed mRNA-miRNA and PPI networks to investigate the impact of 3′UTR alterations on critical miRNAs during *L. vannamei* development. To conclude, this study presents a comprehensive analysis of the dynamic APA patterns and underlying mechanisms in *L. vannamei* development. Based on our current knowledge, this work represents the first systematic investigation of the functional implications of APA during *L. vannamei* development.

## Data Availability

Data will be made available on request.

## Supplementary Materials

The following supporting information can be downloaded at: https://www.mdpi.com/article/10.3390/genes15070837/s1, Table S1: APA event PDUI values at each time point; Table S2: GO enrichment results.
